# Why Movement Is Captured by Music, but Less by Speech: Role of Temporal Regularity

**DOI:** 10.1371/journal.pone.0071945

**Published:** 2013-08-02

**Authors:** Simone Dalla Bella, Anita Białuńska, Jakub Sowiński

**Affiliations:** 1 Movement to Health Laboratory (EuroMov), University of Montpellier-1, Montpellier, France; 2 Institut Universitaire de France, Paris, France; 3 Department of Cognitive Psychology, University of Finance and Management, Warsaw, Poland; University of Milan, Italy

## Abstract

Music has a pervasive tendency to rhythmically engage our body. In contrast, synchronization with speech is rare. Music’s superiority over speech in driving movement probably results from isochrony of musical beats, as opposed to irregular speech stresses. Moreover, the presence of regular patterns of embedded periodicities (i.e., meter) may be critical in making music particularly conducive to movement. We investigated these possibilities by asking participants to synchronize with isochronous auditory stimuli (target), while music and speech distractors were presented at one of various phase relationships with respect to the target. In Exp. 1, familiar musical excerpts and fragments of children poetry were used as distractors. The stimuli were manipulated in terms of beat/stress isochrony and average pitch to achieve maximum comparability. In Exp. 2, the distractors were well-known songs performed with lyrics, on a reiterated syllable, and spoken lyrics, all having the same meter. Music perturbed synchronization with the target stimuli more than speech fragments. However, music superiority over speech disappeared when distractors shared isochrony and the same meter. Music’s peculiar and regular temporal structure is likely to be the main factor fostering tight coupling between sound and movement.

## Introduction

Music compels us to move. When spontaneously tapping our feet or swaying our body along with our preferred song, while dancing, or performing synchronized sports (e.g., swimming), we entrain to the regular pulse and rhythm of music. This propensity to coordinate movement with music, mostly during group activities, transcends places and cultures [1,2]. Not surprisingly, indeed, music is perfectly suited to act as a coordinating device at a group level. Because of its communal character, synchronization with music is thought to foster social bonding [3–6], thus favoring socially-rooted behaviors that are very distinctive of our species. Indeed, some species such as crickets and fireflies provide spectacular examples of synchronization with external rhythmical stimulation in nature [7–9]. Moreover, bird species which are vocal learners can to a certain extent couple their movements to musical beat [10,11]. Nevertheless, humans exhibit unique flexibility in their ability to achieve synchrony with an external timekeeper [12–14].

From early infancy, humans show sensitivity to rhythmic properties of auditory stimuli. They react to violations in repetitive timing patterns (i.e., meter [15–17]), and can code meter in auditory patterns via body movement [18]. Based on this precocious ability to extract regular temporal patterns (e.g., the underlying pulse), 2.5-year-old children start adjusting their movements to the beat of an auditory stimulus, in particular when interacting with a social partner [19,20]. This tie between movement and musical rhythm is probably originating in the first infant–mother interaction [21]. Coupling movement to an external auditory rhythm is supported by a dedicated neuronal network involving both subcortical areas (e.g., the basal ganglia and the cerebellum) and cortical regions (e.g., temporal cortex, premotor regions, and the Supplementary Motor Area) [22–24]. In sum, the pervasive tendency to couple movement to musical beats is a human trait with a defined neuronal substrate which may have played an important role in the origin of music [1,25].

The ubiquity of synchronization with music contrasts with the lack of spontaneous motor synchronization with other complex auditory stimuli, such as spoken utterances. Speech, albeit featuring rich rhythmic organization [26–29] and serving as an inter-personal communication device [30], unlike music, is typically not well suited for synchronized movement. Thus, it is not surprising that there is a paucity of studies on synchronization with speech material. In the only study to date devoted to this question, high inter-tap variability shown by a coefficient of variation around 30% of the average inter-tap interval was reported when participants synchronized with French and English spoken sentences [31]. This performance contrasts with markedly lower coefficients of variation when synchronizing with music (around 4% of the inter-tap interval) [32]. One of the reasons for these differences may lie in the regularity of musical beats (i.e., isochrony) as opposed to speech stresses. Regular isochronous beats are a universal property of music defining its rhythm [33,34], and affording a synchronized motor response [35–37]. Beat perception is supported and reinforced by the properties of the musical structure, characterized by temporal patterns with multiple embedded periodicities [37,38]. These patterns result in the perception of predictable sequences of strong (accented) and weak beats. For example, different meters distinguish marches (characterized by a strong-weak binary pattern) from waltzes (with a strong-weak-weak ternary pattern).

Stresses in speech similarly evoke a subjective impression of isochrony [39]. Yet, the notion of periodicity for speech rhythm (e.g., in stress-timed and syllable-timed languages, like English and French, respectively [40,41]) did not find empirical support, at least in the case of conversational speech [28,42–44]. Inter-stress-intervals are typically highly variable in speech, with coefficients of variations greater than 30% of the average inter-stress-interval [31,43]. These values are typically larger than the variability of inter-beat-intervals observed in performed expressive music, that shows coefficients of variation between 10% and 30% [45]. Moreover, metrical phonology, in analogy with metrical approaches to rhythm in Western Music [46], has similarly proposed a hierarchical metrical structure in speech, based on rhythmic prominence of linguistic units (i.e., syllables, words, and phrases) [26,47]. Nevertheless, speech meter in conversational speech is clearly less strict and regular (i.e., weaker) than musical meter (see 28 for a discussion). Higher regularity is found in poetry [48–50], and speech production in group such as prayers and chanting (i.e., choral speaking [51]). These manifestations of speech can be generally referred to as “metrical speech”. In sum, apart from metrical speech, it appears that speech mostly misses fundamental rhythmic properties, commonly present in music, such as a predictable regular beat and metrical structure, needed to drive synchronized movement.

Why does music typically have a stronger pull than speech on motor synchronization? Music, because of its greater temporal regularity, may be better suited than speech to recruit domain-general mechanisms responsible for extracting beat/stress patterns from a complex acoustic signal. Potential candidates for such mechanisms are cognitive processes supporting entrainment of attention to the temporal properties of auditory sequences (e.g., musical beats or stress patterns in speech) [35], or more low-level mechanisms treating acoustic features relevant for rhythm perception (e.g., amplitude envelope rise time) [52]. This possibility entails that speech utterances displaying music-like temporal patterns (i.e., with regular beat and metrical structure; for example, metrical speech) should attract movement as well as music does. Yet, temporal regularity may not be sufficient alone to account for this effect. The alternative hypothesis is that beat extraction and synchronization to music may require dedicated processes which are music-specific. Indeed, additional cues inherent in the musical structure, engaging domain-specific processes, such as pitch relationships may also favor synchronization. Melodic accents are another source responsible for our perception of meter. This possibility is in keeping with the *joint accent structure* hypothesis [53–57], implying that musical rhythm results from a multilayered structure of relationships among features, such as durations and pitch. A direct consequence is that music may still foster motor synchronization more than metrical speech, in spite of the fact that both share a regular temporal structure. These possibilities have not been examined so far. Moreover, in general, evidence is scant on the comparison of speech and music with regard to synchronization, in spite of its potential interest for clarifying whether beat/meter processing is supported by domain-specific or rather by general-purpose mechanisms.

To examine the role played by temporal regularity (i.e., beat isochrony and meter) on sensorimotor synchronization in music and speech in the present study, we conducted two experiments using the synchronized tapping task. Sensorimotor synchronization has been mainly examined by asking participants to tap their index finger in correspondence with isochronous stimuli, which lack the temporal complexity of music and natural speech (for reviews, see 24,58,59). The tapping paradigm has been quite extensively applied to synchronization with music (e.g., [32,57,60], and more recently to speech stimuli [31,61]. In our experiments we adopted a distractor paradigm [62,63]. Participants are asked to tap their finger along with an isochronous sequence (i.e., a metronome) while periodic distractors (e.g., another isochronous sequence, in the same or in a different modality) are presented at one of various temporal offsets [64–66]. Movement attraction by the distractor is reflected by systematic modulation of the asynchronies (i.e., the relative phase) between the taps and the target sounds, and of the variability of these asynchronies. The magnitude of the systematic change in relative phase and its variability are indicative of the distractors’ degree of interference. The distractor paradigm was successfully adopted to show that rhythmic movement is attracted more strongly to auditory than to visual rhythms [63], but see 64. Moreover, it was shown that asynchrony is typically more negative in the presence of leading distractors and less negative (or more positive) in the presence of lagging distractors [63,65]. Since in this paradigm the distractors are to be ignored, their disrupting effect on synchronization indicates an irresistible tendency of the distractors to capture participants’ movement.

In the two experiments, the effects of music and metrical speech distractors on synchronization with a metronome were compared. The temporal structure of spoken utterances (i.e., examples of metrical speech) was manipulated. Those manipulations pertained to duration and were meant to enhance speech temporal regularity, so that the utterances progressively matched music material in terms of beat/stress isochrony (Exp. 1) and the associated metrical structure (Exp. 2). In Exp. 1 we examined whether beat isochrony embedded in a speech stimulus is less effective in attracting movement as compared to a musical context. Music distractors were computer-generated fragments of familiar music. Speech distractors were spoken fragments of familiar children poetry, chosen for their regular stress pattern and regular metrical structure, and thereby natural conduciveness to synchronized movement. Additional stimulus manipulations were carried out to attain maximum comparability between speech and music. Since in the original stimuli pitch separation between the target sounds and the distractors was smaller for music than for speech, the distractors were equalized in terms of average fundamental frequency (pitch height). Average pitch height was controlled, in so far as phase correction mechanisms underlying distractor effects are not completely insensitive to pitch differences [66]. In another condition, even though speech distractors displayed very regular beats, with minor deviations from isochrony, inter-stress-intervals were additionally manipulated to achieve perfect isochrony, like in music distractors. If music has a greater pull than speech on motor synchronization exclusively because of beat isochrony, we predict that by equalizing beat/stress isochrony the differences between the two domains should disappear. In contrast, if domain-specific musical features play a role, music should still interfere more that metrical speech with synchronization to a target stimulus.

In Exp. 2, new participants were asked to synchronize with isochronous target stimuli while one of three types of distractors was presented in a distractor paradigm. Both music and speech distractors were derived from well-known songs, and performed by a professional singer without accompaniment. Renditions of the songs performed with lyrics, using only the repeated syllable /la/, and the metrically spoken lyrics were used as distractors. Stimuli were manipulated so that inter-beat-intervals and inter-stress-intervals were equally isochronous. Moreover, the duration of corresponding events (i.e., syllables and notes) in between musical beats and linguistic stresses was equalized. Hence, speech and music distractors shared beat/accent isochrony as well as the same metrical structure. If factors beyond temporal regularity (e.g., pitch relationships) contribute to explain music’s greater tendency to favor synchronized movement, music should still attract movement more than metrical speech.

## EXPERIMENT 1

### Materials and Methods

#### Participants

Three groups of native Polish-speaking students without formal musical training from the University of Finance and Management in Warsaw took part in the study in exchange for course credits: Group 1 (*n* = 38, 29 females, mean age = 24.8 years, range = 19-52 years, 36 right-handed and 2 left-handed), Group 2 (*n* = 30, 27 females, mean age = 22.6 years, range = 20-31, 29 right-handed and one left-handed), and Group 3 (*n* = 30, 25 females, mean age = 22.4 years, range = 19-39, 26 right-handed, 4 left-handed). None of the participants reported hearing disorders or motor dysfunction.

#### Material

We used a Target sequence formed by thirty-five 30-ms computer-generated tones with constant pitch (880 Hz sinusoids with a linear 17-ms down-ramp) and intensity presented with an inter-onset interval (IOI) of 600 ms. The Music distractors were three computer-generated well-formed musical fragments from familiar music written in binary meter (i.e., circus music, “Sleighride”, and Bee Gees’ “Stayin’ Alive”) including 29 to 33 musical beats (inter-beat-interval = 600 ms). Speech distractors were well-formed fragments with 28 to 32 stresses from three well-known excerpts of Polish children poetry („Pstryk” and „Lokomotywa” [67]; „Na straganie” [68]). „Pstryk” and „Na straganie” were written in a binary meter (i.e., every second syllable was stressed), „Lokomotywa” in a ternary meter (i.e., every third syllable was stressed). Note that Polish is usually described as a stress-timed language (with lexical stress occurring on the penultimate syllable). Yet, the classification of Polish in terms of rhythm is still quite controversial, suggesting that it should be placed in between stress-timed and syllable-timed languages [93,94]. Speech fragments were read by an actor who was instructed to utter the sentences using adult-directed speech while synchronizing speech stresses to the sounds of a metronome (IOI = 600 ms), and recorded. The mean inter-stress-interval of the recorded speech fragments was 598 ms (*SD* = 66 ms), indicating that the actor was able to maintain the speech rate, as instructed. Distractor stimuli were all normalized to the same maximum intensity level. This condition is referred to as *Original*. Distractors’ familiarity was assessed by asking 32 additional students (28 females, mean age = 20.6 years, range 20-28 years) to rate the distractors on a 10-point scale (1 = not familiar; 10 = very familiar). Music and speech distractors did not differ in terms of familiarity (for music, mean rating = 6.7; for speech, mean rating = 7.0; *t* < 1).

In the *Pitch* condition, the music and speech stimuli were equalized in terms of average pitch, to ensure that this variable did not affect or bias the tendency of the two distractors to pull synchronization. This manipulation was motivated by the observation in previous studies that the pitch of the distractor (e.g., in isochronous sequences) can affect synchronization with a target sound. For example, low distractors tend to exert stronger attraction than high distractors ( [66], but see the same study, Exp. 2, for the opposite effect). Music distractors were manipulated so that their average fundamental frequency (130.6 Hz, *SD* = 17.6 Hz) was comparable to the average fundamental frequency of speech distractors (130.0 Hz, *SD* = 30.0 Hz). Fundamental frequency was computed with Praat software [69] using autocorrelation [70]. This manipulation was achieved by transposing the entire musical excerpt so that its average fundamental frequency matched that of speech distractors. Pitch range and intervals were not manipulated. In the *Pitch+Timing* condition, speech distractors were additionally manipulated with Audition 1.5 software (Adobe, Inc.), to obtain exact 600-ms inter-stress-intervals. The time of occurrence of speech stresses was estimated by listening to the stimuli and by concurrent visual inspection of sounds’ waveform and spectrogram using Praat software. This manipulation (8.7% of the inter-stress-interval, on average), obtained by linear stretching or compressing the waveform between two subsequent speech stresses, did not engender unnatural or abrupt tempo changes (as judged by the experimenters), thus attesting that inter-stress-intervals were already quite isochronous in the original stimuli. In a few cases, stretching segments of the waveform led to acoustic artifacts (e.g., clicks), which were manually removed. In addition, 6 nonmusicians were asked to rate all the stimuli in terms of naturalness on a scale from 1 (= not artificial) to 6 (very artificial). After manipulation, music and speech distractors did not sound more artificial (mean ratings = 2.66 and 2.86, respectively) than in the original condition (mean ratings = 2.21 and 2.51), as attested by non-parametric Wilcoxon tests.

**Figure 1:**
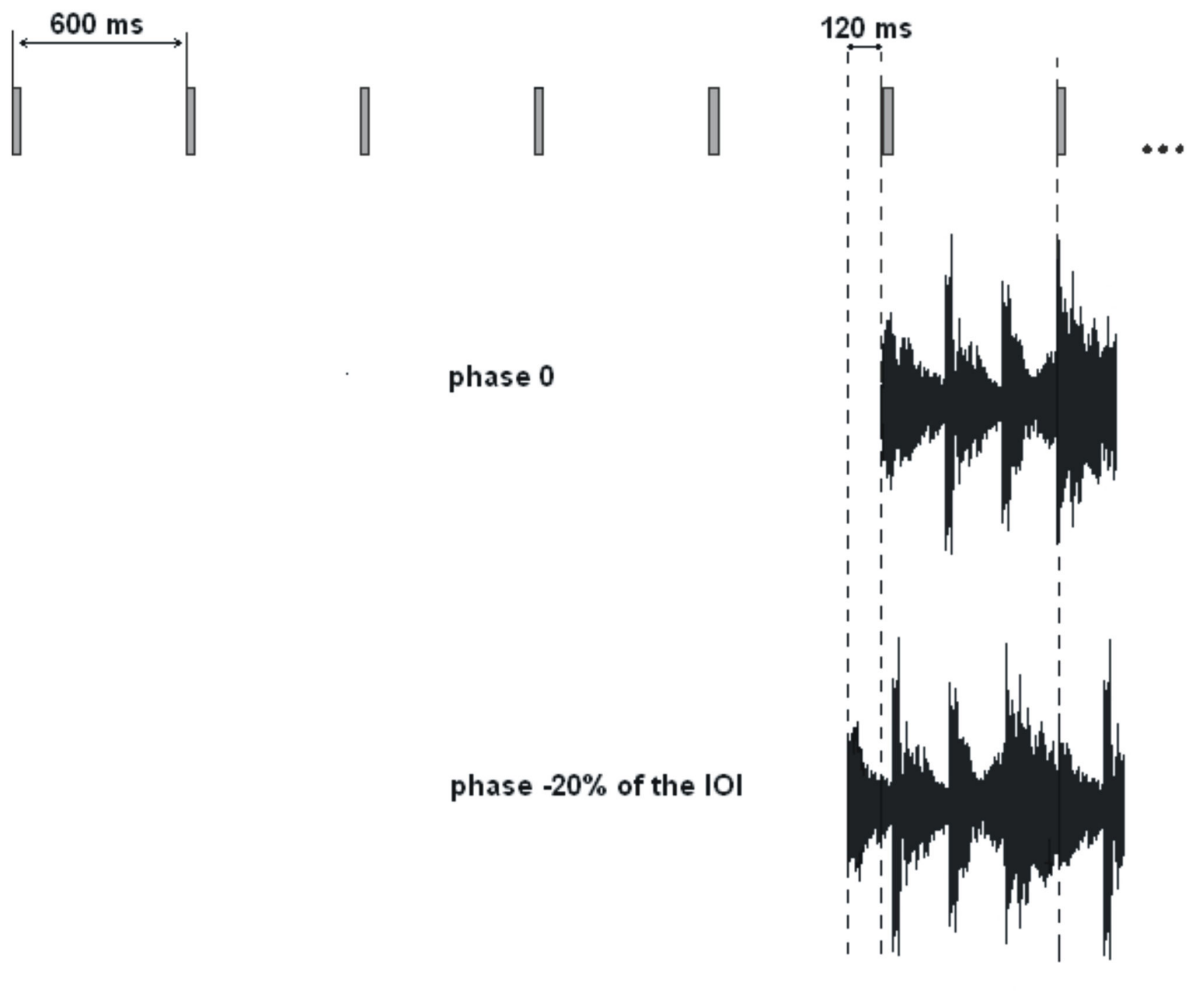
Examples of target-distractor alignment at phase 0 (target sounds and musical beats or speech stresses occur at the same time) and at phase -20% of the IOI (i.e., with musical beats or speech stresses occurring 120 ms before target sounds).

In all conditions, after the first five sounds of the target sequence, the distractor was presented at a particular temporal separation from the sixth target sound. The first musical beat/speech stress of distractor stimuli was determined by the experimenters by visual inspection of the waveform and by listening to the stimuli. Twenty relative temporal separations (i.e., “relative phases”, called hereafter simply “phases”) were used. At phase 0 the sixth sound of the target sequence and the first musical beat or speech stress of the distractor occurred at the same time (for an example, see Figure 1; sound examples can be found at http://www.mpblab.vizja.pl/dallabella_et_al_plos1_stimuli.html). The sixth sound of the target sequence was aligned with the time of occurrence of the first musical beat or of the first speech stress. Target-distractor alignment at phase 0 led to comparable synchronization performance with musical and speech stimuli. This was ascertained in a pilot experiment with 17 nonmusicians. The participants who did not take part in the main experiments were asked to synchronize the movement of their index finger to the target sequence with music or speech distractors presented at phase 0. The remaining 19 phases ranged from -50% of the IOIs (-300 ms) to +45% of the IOIs (+270 ms) with a step of 5% of the IOIs (30 ms). Negative and positive phases indicate that the musical beats and speech stresses occurred before and after target sounds, respectively (see Figure 1). Musical stimuli were generated with a Yamaha MidiRack synthesizer. Speech sequences were recorded with a Shure SM58 microphone onto a hard-disk through Fostex D2424 LV 24 Track Digital Recorder (sampling rate = 44.1 KHz). Stimulus manipulations were carried out using a PC-compatible computer.

#### Procedure

Each group was assigned to one condition (i.e., Group 1 to the *Original* condition, Group 2 to the *Pitch* condition, and Group 3 to the *Pitch+Timing* condition). Participants, sitting in a quiet room in front of the computer monitor, were asked to tap the index finger of their dominant hand along with the sounds of the target sequence alone (*Target only*). In further tasks, they synchronized with the same target sequence while music distractors, or speech distractors were presented. Participants were explicitly instructed to try to ignore the distractor. Targets and distractors were presented binaurally over Sennheiser eH2270 headphones at the same comfortable intensity level. Motor responses were recorded with a tapping pad with 1-ms accuracy built for the purpose of this experiment. The tapping pad provided auditory feedback at the time of the tap, due to the contact of the pad with the tabletop. The experiment was run on Presentation software (Neurobehavioral Systems, Inc.) using a PC-compatible computer. For each distractor type (i.e., music or speech) there were three blocks of trials, one for each of the three stimuli. In one block the distractor+target stimuli sequences at all phases were presented in random order. The *Target only* condition was performed twice, before performing the conditions with distractors. The order of the distractors (i.e., music or speech) and the order of the blocks were counterbalanced across subjects. The experiment lasted approximately 1 hour and a half.

#### Ethics statement

The study was approved by the Ethics Committee of the University of Finance and Management in Warsaw. Written informed consent was obtained from all participants.

### Results and Discussion

Data were first analyzed to ensure that the perturbation of tapping due to music and speech distractors followed a pattern across phases which is comparable to the one observed in previous studies with simpler distractors (i.e., isochronous sequences [63]). To this aim, for each tapping trial, the signed time differences between the target sounds and the taps were computed (as in [63]). These differences are referred to “*signed asynchronies*”. Mean signed asynchrony for each tapping trial was computed for data in the *Target only* and in the *Original* conditions, and submitted to the following analyses. By convention, signed asynchrony is negative when the tap precedes the target sound, and positive when the tap is delayed. The *SD* of the time differences between the targets sounds and the distractors was also calculated (*SD of asynchrony*), as a measure of synchronization variability.

Five out of 98 participants were discarded based on the results obtained in the *Target only* condition: they produced less than 23 consecutive synchronized taps (80% of the maximum number of taps) and exhibited high variability (the *SD* of the asynchrony between target sounds and the taps was larger than 10% of the IOI). Taps corresponding to the first 7 target sounds were not analyzed, as in [63]. Signed asynchrony in the *Target only* condition did not significantly differ across groups, indicating comparable synchronization accuracy (Group 1, signed asynchrony = -46.3 ms, *SD* asynchrony = 34.8 ms; Group 2, async. = -50.0 ms, *SD* async. = 32.9 ms; Group 3, async. = -51.4 ms, *SD* async. = 30.8 ms).

**Figure 2:**
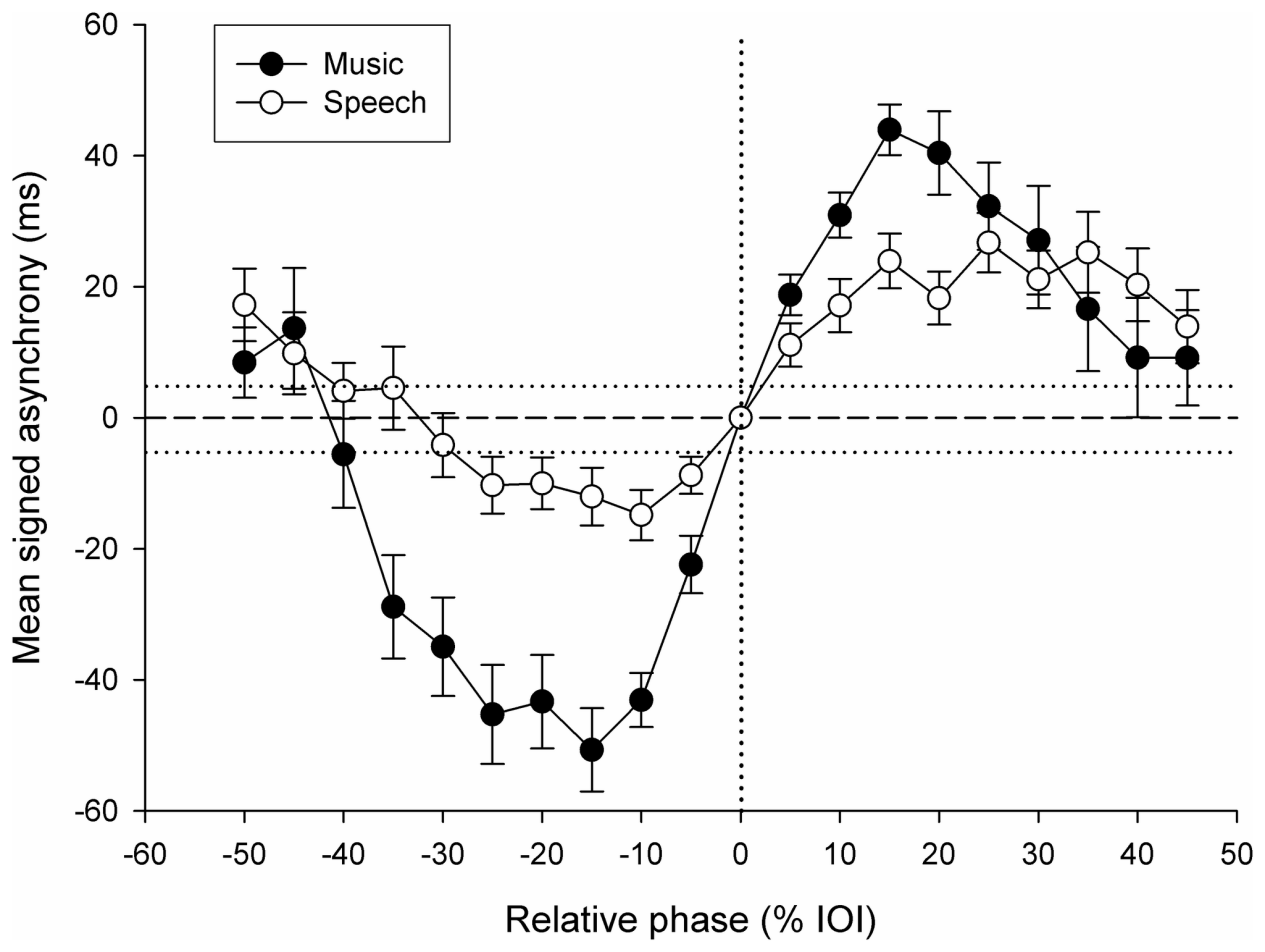
Exp. 1: mean signed asynchrony with music and speech distractors as a function of the relative phase between distractors and target sounds in the *Original* condition. Error bars indicate *SE* of the Mean. The horizontal dotted lines around 0 asynchrony (dashed line) indicate ± *SE* of signed asynchrony obtained in the *Target only* condition.

Mean signed asynchronies were computed at each of the 20 phases. At phase 0 (baseline), where no interference was expected, signed asynchrony was negative, and comparable across music and speech distractors (= -46.9 ms with music distractors and -47.7 ms with speech distractors; *t* < 1). This confirms the anticipation tendency (i.e., mean negative asynchrony) typically observed in sensorimotor synchronization [71]. Data were aligned with respect to the baseline by subtracting asynchrony at phase 0 (averaged separately for each distractor type) from the mean signed asynchronies obtained at all relative phases for the same distractor. Mean signed asynchrony with music and speech distractors in the *Original* condition is illustrated in Figure 2, as a function of the phase of the distractor. Zero signed asynchrony corresponds to the same asynchrony obtained at phase 0. Negative signed asynchrony indicates that the distractor typically increased negative asynchrony from the target stimulus with respect to the baseline; positive signed asynchrony indicates that the distractor reduced negative asynchrony as compared to phase 0, and sometimes (i.e., at larger deviations) led to positive asynchrony. Both music and speech distractors affected synchronization beyond normal tapping variability, as attested by several points in the asynchrony curves falling out of the confidence interval (i.e., 0 ± standard error of asynchrony obtained in the *Target only* condition) represented in Figure 2 by horizontal dotted lines. This pattern of responses, showing the highest perturbation of synchronization around 20-30% of the IOI is consistent with previous studies using isochronous sequences as distractors [63]. Hence, the distractor paradigm can be extended to more complex sequences, such as speech and music. Moreover, since both music and speech showed a similar perturbation profile across phases, the direction of asynchrony was not further considered in the following analyses, and data from different phases were merged before comparing the degree of perturbation caused by the two distractors (see below).

**Figure 3:**
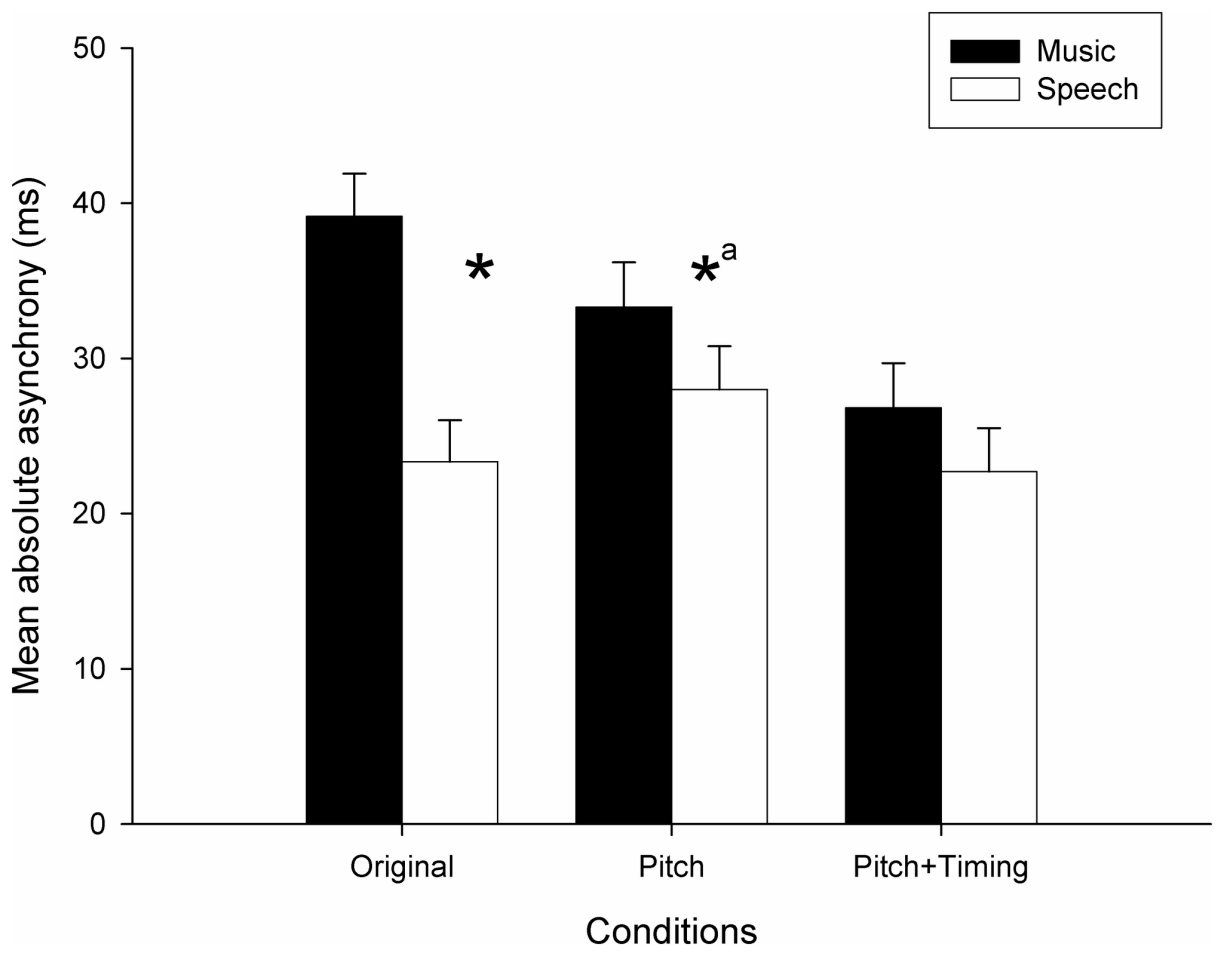
Exp. 1: mean absolute asynchrony obtained in the *Original*, *Pitch*, and *Pitch+Timing* conditions, for music and speech distractors. Error bars are *SE* of the Mean. Stars indicate significant differences (*a* = marginally significant).

To measure the degree of perturbation induced by music and by speech, irrespective of the direction of the asynchrony (i.e., whether it was positive or negative), *absolute asynchrony* was computed. This measure, more parsimonious than relative asynchrony and more appropriate to compute synchronization error, was obtained by taking the absolute values of signed asynchrony at all phases (except at phase 0, where deviation was obviously 0) and by computing their average. Mean absolute asynchrony for *Original*, *Pitch*, *Pitch+Timing* conditions and for music and speech distractors is reported in Figure 3. Absolute asynchronies were entered in a 3(condition) x 2(distractor) mixed-design ANOVA, considering subjects as the random variable. Condition (original vs. pitch vs. pitch+timing) was the between-subjects factor, and Distractor (music vs. speech) was the within-subjects factor. Data from phase 0 were not entered in the ANOVA, because absolute asynchrony in this case was always 0. Music distractors interfered with synchronization more than speech distractors, but this effect was not observed in all conditions, as attested by a significant Condition x Distractor interaction (*F*(2,90) = 4.67, *p* < .05). Music interfered more than speech in the *Original* condition (*F*(1,90) = 29.10, *p* < .001); in the *Pitch* condition, the difference between distractors was only marginally significant (*F*(1,90) = 2,99, *p* = .09). The effect of the distractors did not differ in the *Pitch+Timing* condition. In addition, to obtain a measure of interference when the distractor preceded the target stimulus (i.e., leading) vs. when the distractor was presented after the target stimulus (i.e., lagging), absolute asynchrony was averaged for all negative phases and for all positive phases, separately. Leading distractors (with asynchrony = 32.9 ms, *SD* = 19.4 ms) were more disruptive than lagging distractors (async. = 28.3 ms, *SD* = 17.7 ms) only in the *Pitch* condition (*t*(29) = 2.26, *p* < .05).

**Figure 4:**
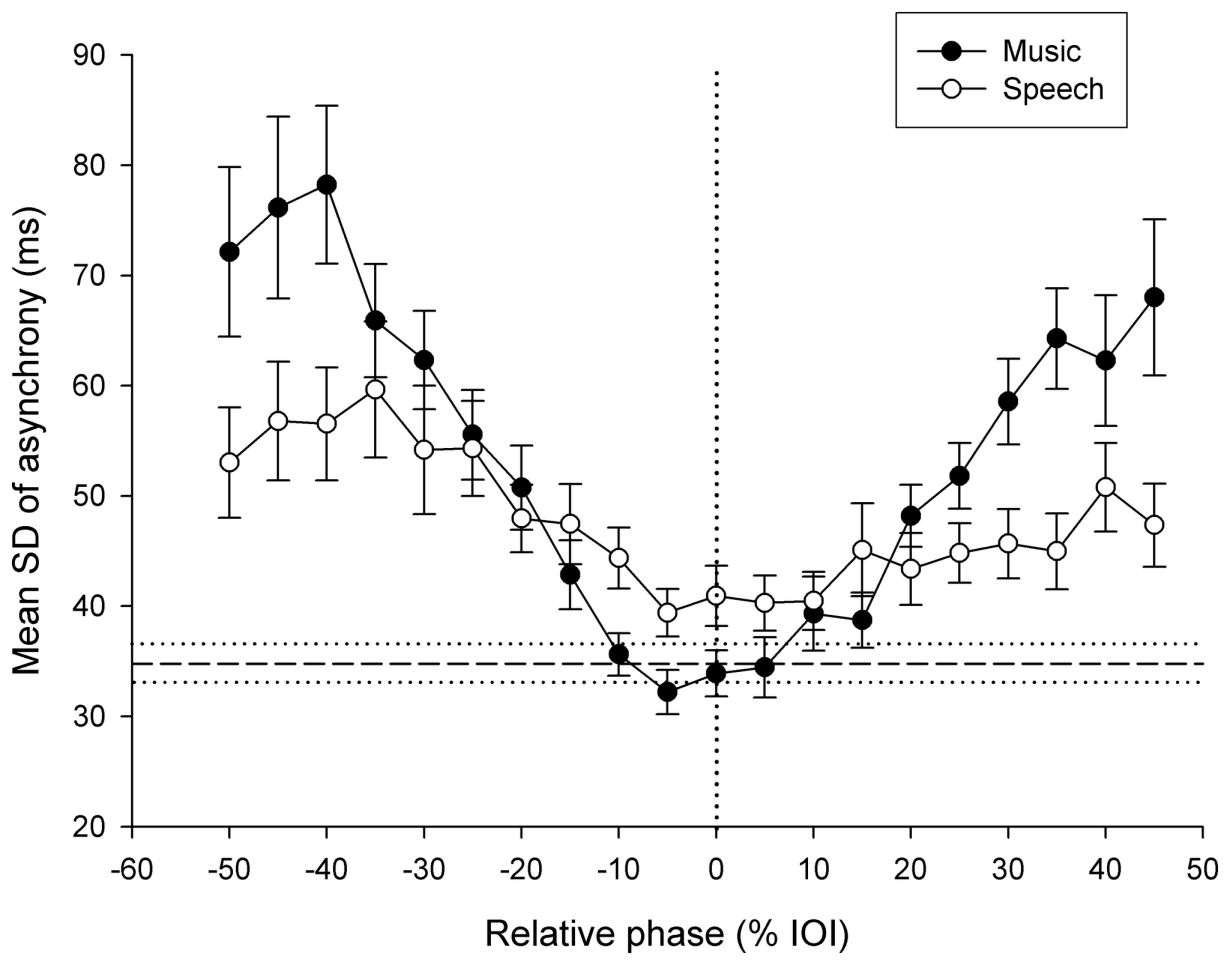
Exp. 1: mean *SD* of asynchrony with music and speech distractors as a function of the relative phase between distractors and target sounds in the *Original* condition. Error bars indicate *SE* of the Mean. The horizontal dotted lines indicate ± *SE* of *SD* of asynchrony obtained in the *Target only* condition.

**Figure 5:**
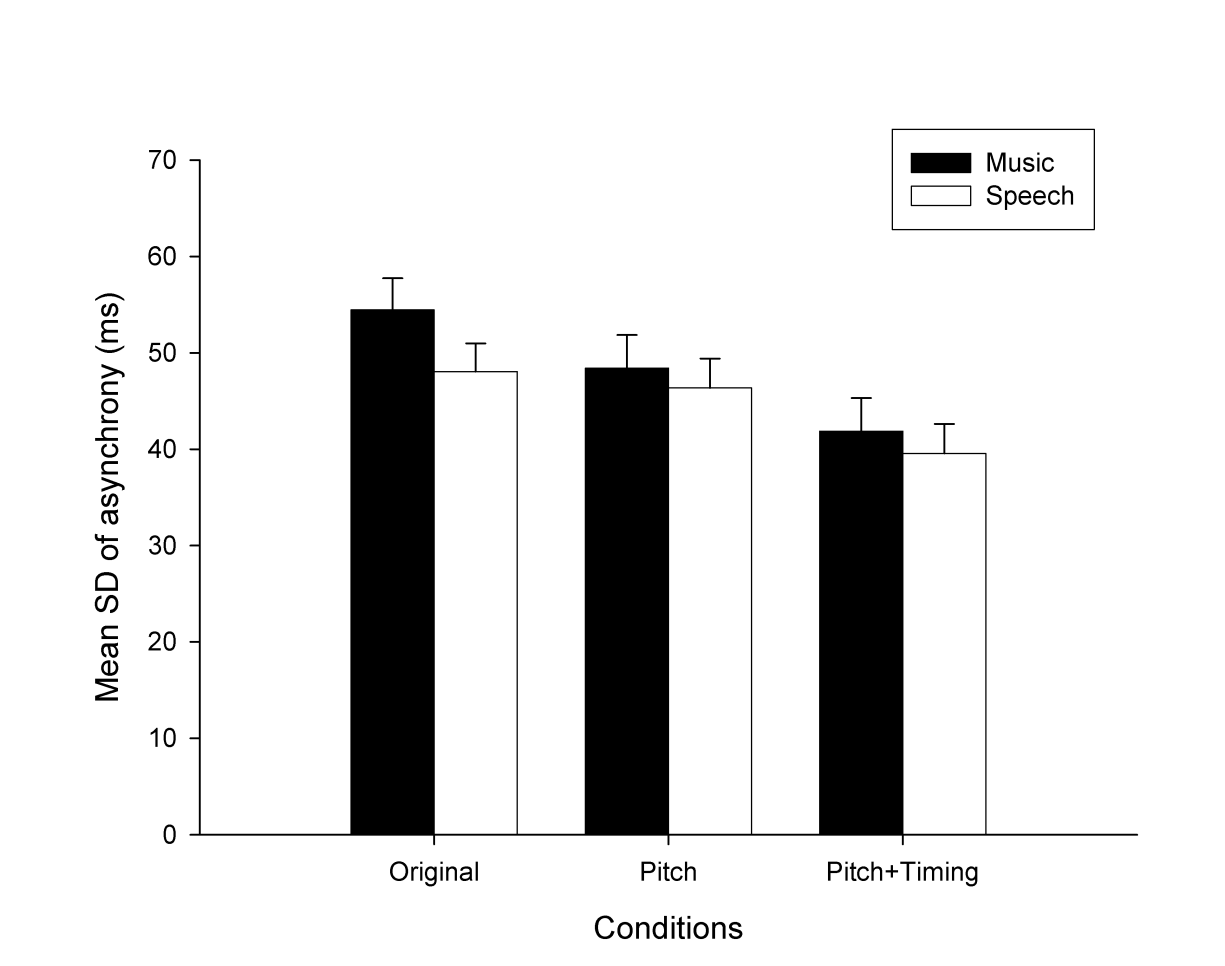
Exp. 1: mean *SD* of asynchrony obtained in the *Original*, *Pitch*, and *Pitch+Timing* conditions, for music and speech distractors. Error bars are *SE* of the Mean.

*SD* of asynchrony was considered to assess whether music and speech distractors differentially affected synchronization variability. This measure at phase 0 (baseline) was larger with speech distractors (*SD* = 35.8 ms) than with music distractors (*SD* = 31.3 ms) (*t*(92) = 3.36, *p* < .01). Mean *SD* of asynchrony with music and speech distractors in the *Original* condition is reported in Figure 4 as a function of the relative phase of the distractor. The distractors induced more variability than observed when participants synchronized with targets alone, as several points in the *SD* of asynchrony curves fell out of the confidence interval (i.e., Mean ± SE for *SD* of asynchrony in *Target only* condition, indicated by the horizontal dotted lines). Mean *SD* of asynchrony for *Original*, *Pitch*, *Pitch+Timing* conditions and for music and speech distractors are reported in Figure 5. Data were entered in a 3(condition) x 2(distractor) mixed-design ANOVA. Greater variability of asynchrony was found with music distractors than with speech distractors across all conditions as indicated by a main effect of Distractor (*F*(1,90) = 8.74, *p* < .01). Moreover, *SD* of asynchrony was progressively smaller in the *Pitch* and *Pitch+Timing* conditions as compared to the *Original* condition (main effect of Condition, *F*(2,90) = 3.13, *p* < .05). The Condition x Distractor interaction did not reach significance. The findings with absolute asynchrony and *SD* of asynchrony were replicated when the ANOVAs were run taking only distractors having a binary meter, namely the three music distractors, and two speech distractors. Hence, meter differences across domains cannot account for the smaller perturbation effect of speech distractors. Finally, leading distractors (*SD* async. = 48.9, *SE* = 2.0) were more disruptive than lagging distractors (*SD* async. = 44.4 ms, *SE* = 1.6) in all three conditions (*t*(92) = 5.94, *p* < .001). These differences between music and speech distractors were confirmed in two additional control experiments, in which 1) the original target sequence (tones) was replaced by a non-musical target sequence (*n* = 30), and 2) when the intensity for each beat/speech stress was normalized to the same maximum intensity level for all distractors (*n* = 34). In these experiments music distractors still led to higher variability of asynchrony than speech distractors did.

In sum, both music and speech distractors perturbed sensorimotor synchronization with the target sequence. Although the taps were attracted to both leading and lagging distractors, the effect was often larger when the distractor preceded the target, consistent with previous evidence [63,65]. Music disturbed synchronization with the target more than speech in the *Original* condition and when average pitch was controlled (i.e., *Pitch* condition). Due to the music distractor, participants were less accurate (i.e., they tapped farther from the target sounds) and were more variable. Interestingly, when the beats/stresses in the two distractors were equally isochronous (i.e., *Pitch+Timing* condition), music still led to increased variability. Yet, the discrepancy between speech and music in terms of absolute asynchrony was no more visible. These findings generally indicate that beat isochrony differently affects synchronization depending on the context in which it is embedded. That music kept disturbing synchronization more than speech even when all distractors shared beat/stress isochrony, suggests that other factors may intervene for explaining this difference between the two domains. In Exp. 1, the recurrent patterns of durations supporting an isochronous beat/stress (i.e., the metrical structure) were not totally comparable in music and speech distractors. In the music distractors, the events occurring in between isochronous beats were precisely timed, thus conferring to these stimuli a regular metrical structure. This was not true for speech distractors, which exhibited a less regular metrical structure, in spite of isochrony, even in the *Pitch+Timing* condition. Moreover, another potential confound is that music was computer-generated whereas speech stimuli were read by an actor. Thus, natural stimulus variability may have partly reduced the effectiveness of the speech distractor. In Exp. 2 speech and music distractors were manipulated so that they were comparable not only in terms of beat/accent regularity but also of their metrical structure. As before, their distracting effect on sensorimotor synchronization with a target sequence was examined.

## EXPERIMENT 2

### Materials and Methods

#### Participants

Twenty-nine native Polish-speaking students (25 females) without formal musical training from the University of Finance and Management in Warsaw volunteered to participate in the experiment in exchange for course credits. They were 24.9 years old on average (range = 19-42 years); 28 were right-handed and one was left-handed. No participants reported hearing disorders or motor dysfunctions.

#### Material and procedure

**Figure 6:**
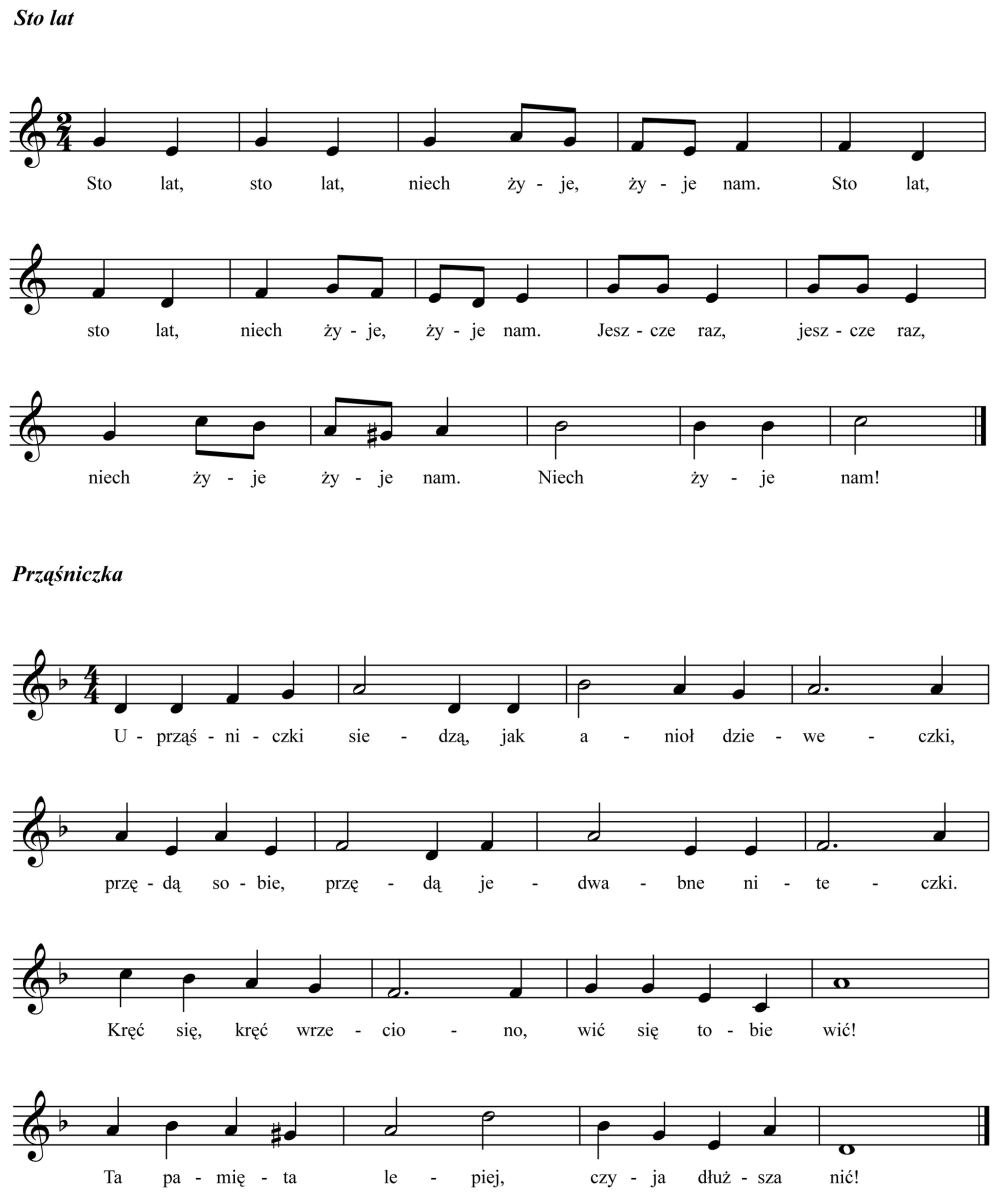
Score of the song stimuli used in Exp. 2.

The procedure and the apparatus are the same as in Exp. 1. The same Target sequence as in Exp. 1 was used. New distractor sequences were created based on two well-known Polish songs („Prząśniczka” and „Sto Lat” [72]), both written in a binary meter (i.e., with beats occurring every second syllable). Two well-formed excerpts of the songs (see Figure 6), including 30 stresses for „Prząśniczka” and 28 stresses for “Sto lat” were used to prepare three novel types of distractors (i.e., *music-lyrics*, *music-syllable*, and *lyrics only* distractors).

The lyrics corresponding to the two song fragments were read by a professional singer with 4 years of formal vocal training, and 21 years of experience as a singer in a professional choir. The singer was instructed to produce speech stresses (i.e., every second syllable) every 600 ms as indicated by a metronome. The metronome sounded through headphones prior to the performance, and was turned off during the recording. The recorded speech fragments were additionally manipulated with Audition software (Adobe, Inc.) so that the inter-syllable onsets for syllables corresponding to musical notes in the song were the same as prescribed by the notation (with inter-stress-interval = 600 ms). As done in Exp. 1, speech stresses were identified by listening to the stimuli and by visual inspection of sounds’ waveform and spectrogram. Finally, the loudness of spoken syllables was equalized by defining the segment around each syllable corresponding to a note in the score (i.e., from -50% of the IOI between the target note and the preceding one to +50% of the IOIs between the target note and the following one), and by normalizing the intensity of each segment to the same maximum intensity level. Whenever acoustic artifacts resulting from this procedure emerged, they were manually removed. As done in Exp. 1, the stimuli were rated in terms of naturalness bv 6 nonmusicians on the same scale used in Exp. 1. In general, speech distractors were rated as more artificial (mean rating = 4.91) than music distractors (mean rating = 3.08). The stimuli thus prepared will be referred to as “lyrics only” distractors.

Music distractors were prepared based on two MIDI piano versions of the two fragments of „Prząśniczka” and „Sto Lat” (inter-beat-interval = 600 ms), with average pitch height corresponding to the average fundamental frequency computed for speech distractors (with Praat software [69]). The average fundamental frequency in MIDI piano versions for „Prząśniczka” was 278.3 Hz (as compared to 271.7 Hz, for the *lyrics only* distractor), and for “Sto lat” it was 313.6 Hz (306.1 Hz, for the *lyrics only* distractor). The same professional singer was asked to sing the two fragments of the songs „Prząśniczka” and „Sto Lat” at the tempo provided by a metronome (IOI = 600 ms) through headphones, and at the pitch height indicated by the MIDI piano version of the fragments. The MIDI file and the metronome indicating musical beats were presented through headphones prior to the performance, and were turned off during the recording. The professional singer performed the melody with lyrics (i.e., for *music-lyrics* distractors), and on the repeated syllable /la/ (i.e., for *music-syllable* distractors). The sung renditions were manipulated so that the note IOIs were the same as prescribed by the notation (with inter-beat-interval = 600 ms). Notes’ loudness was equalized to the same maximum intensity level used for speech distractors. The distractors were further analyzed in terms of amplitude envelope rise times at the moment of the beat/stress, since this dimension is an important cue to speech rhythm [52]. Rise times were longer in music distractors (on average, 289 ms for *music-lyrics*, and 279 for *music-syllable* distractors) than in *lyrics only* distractors (on average, 195 ms). The resulting abrupt changes of amplitude in *lyrics only* distractors may have conveyed a stronger sense of rhythm.

As done in Exp. 1, the target sequence was combined with distractors to obtain 20 sequences with various phases, ranging from -50% of the IOIs to +45%, with a 5% step. There were three blocks, one for each of the distractor types. Each participant was first asked to synchronize with the target sequence alone (*Target only*). The target sequence was then presented with the *music-lyrics*, the *music-syllable*, and the *lyrics only* distractors (for sound examples, see http://www.mpblab.vizja.pl/dallabella_et_al_plos1_stimuli.html). Participants were instructed to tap the index finger of their dominant hand along with the sounds of the target sequence trying to ignore the distractor. The order of distractors was counterbalanced across subjects.

#### Ethics statement

The study was approved by the Ethics Committee of the University of Finance and Management in Warsaw. Written informed consent was obtained from all participants.

### Results and Discussion

Four out of 29 participants were discarded based on the same criteria adopted in Exp. 1. As observed in Exp. 1, signed asynchronies at phase 0 (baseline) were negative and comparable across the three types of distractor (-51.6 ms, with *music-lyrics*, -51.9 ms with *music-syllable*, and -55.4 ms with *lyrics only*; *F* < 1). Hence, data were aligned with respect to the baseline by subtracting average asynchrony obtained at phase 0 for each distractor type from signed asynchronies at all phases for the same distractor.

**Figure 7:**
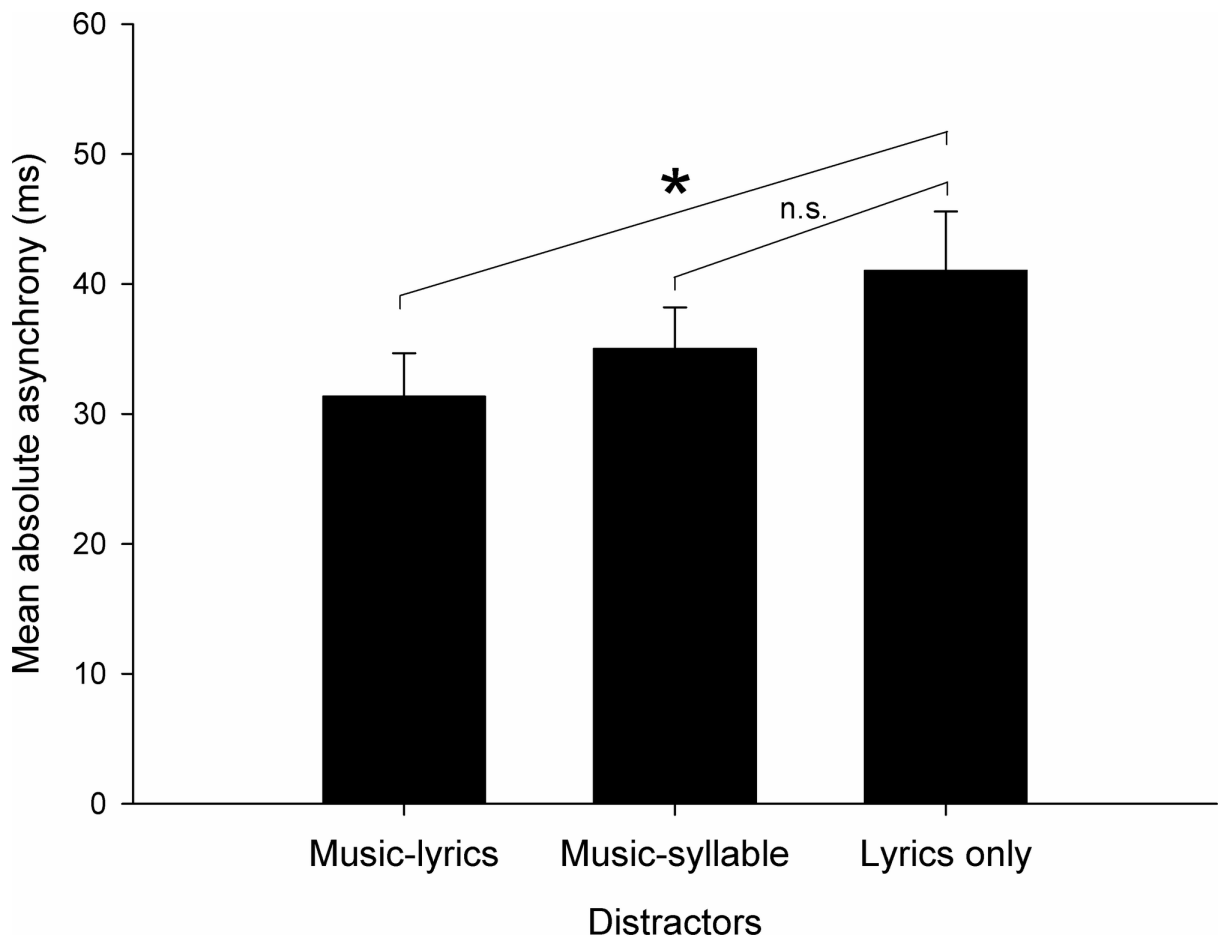
Exp. 2: mean absolute asynchrony for *music-lyrics*, *music-syllable*, and *lyrics only* distractors. Error bars are *SE* of the Mean. The star indicates a significant difference as revealed by a Bonferroni post-hoc test (*p* < .05).

As done in Exp. 1, the degree of perturbation caused by the different distractors was obtained by computing absolute asynchrony for the three distractor types (see Figure 7). These data were entered in a repeated-measures ANOVA, taking subjects as the random variable, and considering Distractor (*music-lyric*s vs. *music-syllable* vs. *lyrics only*) as the within-subjects factor. Distractors had different effects on synchronization (*F*(2,46) = 5.55, *p* < .01). Bonferroni Post-hoc comparisons indicated that *music-lyrics* distractors were less disturbing than *lyrics only* distractors (*p* < .05; see star in Figure 7); the performance with *music-syllable* and *lyrics only* distractors did not significantly differ. In addition, leading distractors (with mean absolute asynchrony = 41.0 ms) were more disrupting than lagging distractors (asynchrony = 30.7 ms) (*t*(23) = 2.72, *p* < .05). *SD* of asynchrony was computed to compare variability in synchronization accuracy with different distractors. *SD* of asynchrony at phase 0 (baseline) was similar with the three distractors (30.8 ms, with *music-lyrics*, 37.7 ms with *music-syllable*, and 32.5 ms with *lyrics only*). Mean *SD*s of asynchrony did not differ as a function of the distractor type (47.0 ms, *SE* = 3.0, with *music-lyrics*; 48.3 ms, *SE* = 3.2, with *music-syllable*; 50.4 ms, *SE* = 3.0, with *lyrics only*). Moreover, no difference was observed between leading and lagging distractors.

Music (i.e., *music-lyrics* and *music-syllable* distractors) did not cause greater disruption of synchronization accuracy (i.e., asynchrony between taps and pacing stimuli and its variability) than speech (i.e., *lyrics only* distractor). Indeed, in one isolated case (when comparing mean absolute asynchrony with *music-lyrics* vs. with *lyrics only* distractors), speech was even more disrupting than music. It is noteworthy that the lack of music superiority over speech in this experiment does not stem from reduced effectiveness of music distractors in Exp. 2 as compared to Exp. 1. Music distractors similarly perturbed synchronization in the two experiments (i.e., in the *music-lyrics* and in the *music-syllable* conditions in Exp. 2, absolute asynchrony = 33.0 ms; *SD* of asynchrony = 47.7 ms; in Exp. 1, across conditions, asynchrony = 34.3 ms; *SD* of async. = 50.3 ms). Instead, the observed difference between music and speech distractors results from greater interference of speech distractors in Exp. 2 (i.e., absolute asynchrony = 41.0 ms; *SD* of asynchrony = 50.4 ms) than in Exp. 1. (i.e., across conditions, asynchrony = 26.3 ms; *SD* of async. = 46.8 ms).

To summarize, having isochrony and the underlying metrical structure embedded in music and speech contexts led to comparable distractor effects; this abolished music superiority over speech in capturing taps. Contrary to our expectations, this finding indicates that auditory stimuli displaying temporal regularity (i.e., an isochronous beat/stress supported by a regular metrical structure) can similarly attract movement in spite of their domain (music or speech).

## Conclusions

In this study we sought to examine whether temporal regularity, in terms of beat isochrony and meter, is uniquely responsible for superiority of music over speech in favoring synchronized motor responses. Using a distractor paradigm, we observed that rhythmic movement is more strongly perturbed by musical beats than by speech stresses (Exp. 1). Participants had more difficulties with tapping in time with a metronome and were more variable when music acted as a distractor than when speech was presented. Making speech more similar to music, by equalizing average pitch and beat/stress isochrony, reduced the discrepancy between the two domains. When both average pitch and isochrony were controlled, speech attracted movement as much as music did (i.e., participants exhibited similar tapping accuracy in the two conditions). Yet, tapping was still more variable with music than with speech in that condition. Hence, both temporal regularity (i.e., the variability of the interval between musical beats/speech stresses), and pitch contributed to explain music superiority in fostering synchronized movement. This finding is in keeping with some evidence that pitch separation between target and distractor sequences may affect the tendency of the distractor to capture movement. Unfortunately, results are quite inconsistent on the role of pitch separation in perturbation as observed in distractor paradigms ( [24], for a review). Yet, in some cases, capture of movement by the distractor has been found more often when pitch separation between target and distractors is smaller ( [65], Experiments 3 and 4; but see 73 for negative results). Note that in Exp. 1, by equalizing music and speech in terms of average pitch, we reduced the pitch separation between the target and the music distractor. This manipulation led to smaller difference between music and speech distractors. In sum, this finding points to a more important role of pitch separation in explaining distractor effects, at least with more complex stimulus material, than suggested by previous studies.

This discrepancy between the two domains completely disappeared when music and metrical speech shared an isochronous beat/stress structure supported by the same meter (Exp. 2). Speech, in some situations, can be as metrical as music, thus similarly favoring synchronized movement. In addition, a particularly puzzling finding is that lyrics alone perturbed synchronized tapping more than sung lyrics. A possible explanation for this finding lays on basic acoustical differences between music and highly metrical speech, like the stimuli used in Exp. 2. Spoken sentences were uttered with a declamatory style, reminiscent of solfege, with the purpose of conveying a clear rhythmical structure. This speaking style is likely to have enhanced acoustical features in the speech signal which are particularly critical for extracting its rhythmical structure. One of such features is amplitude envelope rise time [52,74,75]. Speech distractors revealed indeed shorter rise times in correspondence of speech stresses (i.e., more abrupt changes of the amplitude envelope), as compared to music distractors. As a result, participants may have been primed to pay particular attention to rhythmical properties of spoken material, thus leading to greater interference. Finally, lowered stimulus naturalness resulting from the manipulation of speech material may have also contributed to enhance the distracting effect of speech stimuli.

Which mechanisms are responsible for the observed effects? The ubiquitous tendency of music to favor movement may suggest, *prima facie*, that music engages domain-specific mechanisms subserving beat entrainment and motor synchronization. This view implies that musical beats attract taps more than any other kind of auditory stimulus having the same metrical complexity and regularity. This was not the case in the present study. Our findings rather point toward an account, in which music taps domain-general mechanisms for beat extraction from a complex acoustic signal. These mechanisms would be similarly engaged by metrical speech, because of its regular stress pattern and a hierarchy of embedded periodicities. This possibility is in line with previous suggestions that similar processes support meter perception in speech and music [29,76,77]. Yet, note that conversational speech does not usually share these rhythmical features with metrical speech. Thus, timing in music and conversational speech may still not be governed by the same shared mechanism. Rhythm-based prediction may be supported by quite different processes in music and conversational speech. Only the former would rely on isochrony and embedded periodicities [28].

Different general-purpose mechanisms can account for movement attraction by distractors. Successful sensorimotor synchronization requires error correction (i.e., phase and period correction [59]). In absence of error correction, taps would at a certain point drift away from the target events, due to error accumulation over time [73]. Error correction has been modeled as a linear process, in which the time of occurrence of the tap is corrected by a constant proportion of the asynchrony between the previous tap and the previous target stimulus [78–80]. The attraction of taps to periodic distractors (i.e., phase attraction) is mediated by such correction mechanisms (e.g., phase correction [65]). Studies with the distractor paradigm indicate that the attraction of taps to distractors is mostly due to temporal integration phenomena [66]. This hypothesis implies that interference depends on absolute temporal separation between the distractors and the target sounds. The distractors occurring in the vicinity of target stimuli (within a fixed temporal window around 120 ms) would tend to be perceptually integrated with the target, thus affecting error correction, and eventually disrupting synchronization [24,63]. This account is not incompatible with cross-modal distractor effects [61] and is in agreement with other findings of auditory dominance in cross-modal perception of timing [81,82]. The results obtained in the present study, showing maximum effect of the distractor around 100 ms (e.g., see Figure 2), are consistent with the previously discussed perceptual integration interval. Nonetheless, the differences between speech and music distractors observed in Exp. 1 are difficult to reconcile with the perceptual integration hypothesis, unless we postulate different windows for temporal integration depending on the stimulus domain, or, more generally, for stimuli with different degrees of metrical regularity.

An alternative hypothesis is that taps and distractors independently attract the taps (i.e., in absence of temporal integration). According to this view, attraction of taps to distractors depends on relative phase (i.e., with maximum interference occurring at a variable temporal separation between target and distractors, as a function of stimulus rate), rather than on absolute temporal separation. Explanations compatible with this general hypothesis come from dynamical system accounts. For example, these accounts are successful in modeling bimanual coordination to different sequences with varying relative phase [83,84]. In these studies relative phase, more than a fixed temporal integration window, is able to predict the amount of phase attraction between two sequences performed bimanually. The role of relative phase was not corroborated in unimanual distractor studies, though, at least for in-phase synchronization (see 66 for a discussion). Another possibility, always stemming from the dynamical systems approach, relies on the idea that tapping along with an isochronous pacing stimulus requires the synchronization of an internal attentional rhythm (e.g., internal oscillation) with the target stimuli (i.e., by entrainment; Dynamic Attending Theory [85,86]). After the presentation of a few isochronous stimuli the internal attentional oscillation adapts to the temporal structure of the pacing sequence so that attentional pulses (i.e., maximum attentional energy) soon coincide with the time of occurrence of the target stimuli. Distractors are likely to compete with the target pacing stimuli in attracting listeners’ attention (i.e., the internal oscillation), in particular when the asynchrony between the two is reduced, thus leading to greater perturbation. It is relevant to this discussion that models including more than one attentional oscillator [35,36,85] can track metrical temporal structures, like those observed in music. Metrical stimuli (i.e., having multiple periodicities) excite a set of coupled oscillators with embedded periodicities, which, due to the coupling, are drawn into a stable relationship with each other. This results in lowered variability of the oscillator at the beat period as compared to simple isochronous sequences; moreover, the stronger (and the more regular) the metrical structure, the lower the variability of the oscillator at the beat period [35,87]. This theory can account for the differences between the effects of music and metrical speech observed in Exp. 1. Indeed, a stimulus with stronger and more regular metrical structure (e.g., music) is likely to excite listeners’ internal oscillations more than a stimulus with an isochronous beat without a regular metrical structure (e.g., speech), thus eventually leading to strong phase attraction. One problem with this account lies in its reliance on relative phase, though. Indeed, as mentioned above, interference as observed in in-phase unimanual synchronization depends on temporal integration mechanisms occurring within a fixed temporal window around the target stimulus. Yet, note that the possibility that relative phase may play a role in phase attraction has been suggested by results with anti-phase synchronization ( [66], Exp. 3). Moreover, pacing stimuli and distractors are likely to independently attract the taps but within a restricted range of attraction [66]. An examination of these possibilities awaits further research.

In sum, music is intimately tied to movement. Humans appear as being naturally endowed to move at the beat of music more than along with speech rhythm. This propensity to entrain to the beat of music mainly results from isochronous beats supported by a regular, temporal structure, characterized by multiple periodicities. This is what makes music (sometimes irresistibly) conducive to synchronized movement. Metrical speech manipulated so as to achieve similar temporal regularity can attract movement. In contrast, conversational speech, lacking such degree of temporal regularity, is not well suited for driving synchronized movement. The extraction of metrical properties from the auditory signal during sensorimotor synchronization engages both auditory and dorsal premotor areas of the brain [88,89]. In particular, dorsal premotor cortex, is likely to be crucial also in auditory-motor integration in the analysis of complex temporal organizations [23]. Music, because of its peculiar and regular beat and metrical structure, is likely to uniquely engage brain circuitries underlying sensorimotor integration, thus favoring tight coupling between sound and movement.

Further studies are in order to examine whether the observed differences between music and speech extend across a variety of music and speech stimuli, and whether this effect covaries with musical expertise. For example, some musical genres (e.g., pop or rock music), given to their prominent metrical structure, are likely to differ from speech more than others (e.g., instrumental Renaissance music), in their tendency to foster synchronized movement. Moreover, speech stimuli typically associated to choral speech (e.g., prayers), because of their temporal regularity, should be akin to music in attracting movement. Finally, the distractor paradigm adopted here is likely to be useful for testing rhythm processing in particular in individuals exhibiting poor synchronization [90–92].

## References

[1] McNeill WH (1995) Keeping together in time: Dance and drill in human history. Cambridge, MA: Harvard University Press.

[2] Nettl B (2000) An ethnomusicologist contemplates universals in musical sound and musical culture. In: Wallin NL Merker B Brown S The origins of music. Cambridge, MA: MIT Press pp. 463–472.

[3] Benzon WL (2001) Beethoven’s anvil: Music in mind and culture. New York: Basic Books.

[4] Hove MJ, Risen JL (2009) It’s all in the timing: Interpersonal synchrony increases affiliation. Soc Cogn 27(6): 949-961. doi:10.1521/soco.2009.27.6.949.

[5] Phillips-Silver J, Aktipis CA, Bryant GA (2010) The ecology of entrainment: Foundations of coordinated rhythmic movement. Mus Percept 28(1): 3-14. doi:10.1525/mp.2010.28.1.3. PubMed: 21776183. 10.1525/mp.2010.28.1.3 PMC3137907 21776183

[6] Wallin NL, Merker B, Brown S (2000) The origins of music. Cambridge, MA: MIT Press.

[7] Buck J, Buck E (1968) Mechanism of rhythmic synchronous flashing of fireflies. Fireflies of Southeast Asia may use anticipatory time-measuring in synchronizing their flashing. Science 159: 1319-1327. doi:10.1126/science.159.3821.1319. PubMed: 5644256. 5644256 10.1126/science.159.3821.1319

[8] Buck J (1988) Synchronous rhythmic flashing of fireflies. II. Q Rev Biol 63: 265-289. doi:10.1086/415929. PubMed: 3059390. 3059390 10.1086/415929

[9] Merker B (2000) Synchronous chorusing and human origins. In: Wallin NL Merker B Brown S The origins of music. Cambridge, MA: MIT Press pp. 315-327.

[10] Patel AD, Iversen JR, Bregman MR, Schulz I (2009) Experimental evidence for synchronization to a musical beat in a nonhuman animal. Curr Biol 19(10): 827-830. doi:10.1016/j.cub.2009.03.038. PubMed: 19409790. 19409790 10.1016/j.cub.2009.03.038

[11] Schachner A, Brady TF, Pepperberg IM, Hauser MD (2009) Spontaneous motor entrainment to music in multiple vocal mimicking species. Curr Biol 19(10): 831-836. doi:10.1016/j.cub.2009.03.061. PubMed: 19409786. 19409786 10.1016/j.cub.2009.03.061

[12] McDermott J, Hauser MD (2005) The origins of music: Innateness, uniqueness, and evolution. Mus Percept *23*(1): 29-59

[13] Patel AD (2006) Musical rhythm, linguistic rhythm, and human evolution. Mus Percept 24: 99-104. doi:10.1525/mp.2006.24.1.99.

[14] Merker BJ, Madison GS, Eckerdal P (2009) On the role and origin of isochrony in human rhythmic entrainment. Cortex 45: 4-17. doi:10.1016/j.cortex.2008.06.011. PubMed: 19046745. 19046745 10.1016/j.cortex.2008.06.011

[15] Bergeson TR, Trehub SE (2006) Infants’ perception of rhythmic patterns. Music Percept 23: 345-360. doi:10.1525/mp.2006.23.4.345.

[16] Hannon EE, Trehub SE (2005) Metrical categories in infancy and adulthood. Psychol Sci 16: 48-55. doi:10.1111/j.0956-7976.2005.00779.x. PubMed: 15660851. 15660851 10.1111/j.0956-7976.2005.00779.x

[17] Winkler I, Háden GP, Ladinig O, Sziller I, Honing H (2009) Newborn infants detect the beat of music. Proc Natl Acad Sci USA 106(7): 2468-2471. doi:10.1073/pnas.0809035106. PubMed: 19171894. 19171894 10.1073/pnas.0809035106 PMC2631079

[18] Phillips-Silver J, Trainor LJ (2005) Feeling the beat in music: Movement influences rhythm perception in infants. Science 308: 1430. doi:10.1126/science.1110922. PubMed: 15933193. 15933193 10.1126/science.1110922

[19] Kirschner S, Tomasello M (2009) Joing drumming: Social context facilitates synchronization in preschool children. J Exp Child Psychol 102(3): 299-314. doi:10.1016/j.jecp.2008.07.005. PubMed: 18789454. 18789454 10.1016/j.jecp.2008.07.005

[20] Provasi J, Bobin-Bègue A (2003) Spontaneous motor tempo and rhythmical synchronisation in 2½- and 4-year-old children. Int J Behav Dev 27: 220–231. doi:10.1080/01650250244000290.

[21] Dissanayake E (2000) Antecedents of the temporal arts in early mother–infant interaction. In: Wallin NL Merker B Brown S The origins of music. Cambridge MA: MIT Press pp. 389-410.

[22] Wing AM (2002) Voluntary timing and brain function: An information processing approach. Brain Cogn 48: 7-30. doi:10.1006/brcg.2001.1301. PubMed: 11812030. 11812030 10.1006/brcg.2001.1301

[23] Zatorre RJ, Chen JL, Penhune VB (2007) When the brain plays music: Auditory-motor interactions in music perception and production. Nat Rev Neurosci 8: 547-558. doi:10.1038/nrn2152. PubMed: 17585307. 17585307 10.1038/nrn2152

[24] Repp BH (2005) Sensorimotor synchronization: A review of the tapping literature. Psychon Bull Rev 12(6): 969-992. doi:10.3758/BF03206433. PubMed: 16615317. 16615317 10.3758/bf03206433

[25] Mithen S (2006) The singing Neanderthals. Cambridge, MA: Harvard University Press.

[26] Liberman M, Prince A (1977) On stress and linguistic rhythm. Ling Inq 8: 249–336.

[27] Patel AD, Daniele JR (2003) An empirical comparison of rhythm in language and music. Cognition 87: B35–B45. doi:10.1016/S0010-0277(02)00187-7. PubMed: 12499110. 12499110 10.1016/s0010-0277(02)00187-7

[28] Patel AD (2008) Music, language, and the brain. New York: Oxford University Press.

[29] Port RF (2003) Meter and speech. J Phon 31: 599-611. doi:10.1016/j.wocn.2003.08.001.

[30] Auer P, Couper-Kuhlen E, Müller F (1999) Language in time. The rhythm and tempo of spoken interaction. New York: Oxford University Press.

[31] Lidji P, Palmer C, Peretz I, Morningstar M (2011) Listeners feel the beat: entrainment to English and French speech rhythms. Psychon Bull Rev 18(6): 1035-1041. doi:10.3758/s13423-011-0163-0. PubMed: 21912999. 21912999 10.3758/s13423-011-0163-0 PMC3219863

[32] Snyder JS, Krumhansl CL (2001) Tapping to ragtime: Cues to pulse finding. Mus Percept 18: 455-490. doi:10.1525/mp.2001.18.4.455.

[33] Drake C, Bertrand D (2001) The quest for universals in temporal processes in music. Ann N Y Acad Sci 930: 17-27. PubMed: 11458828. 11458828 10.1111/j.1749-6632.2001.tb05722.x

[34] Stevens C, Byron T (2009) Universals in musical processing. In: Hallam S Cross I Thaut M Oxford Handbook of Music Psychology. New York: Oxford University Press pp. 14-123.

[35] Large EW, Jones MR (1999) The dynamics of attending: How people track time-varying events. Psychol Rev 106(1): 119-159. doi:10.1037/0033-295X.106.1.119.

[36] Large EW, Palmer C (2002) Perceiving temporal regularity in music. Cogn Sci 26: 1-37. doi:10.1207/s15516709cog2601_1.

[37] London J (2004) Hearing in time. Psychological aspects of musical meter. New York: Oxford University Press.

[38] Lerdahl F, Jackendoff R (1983) A generative theory of tonal music. Cambridge, MA: MIT Press.

[39] Lehiste I (1977) Isochrony reconsidered. J Phon 5: 253-263.

[40] Abercrombie D (1967) Elements of General Phonetics. Chicago: Aldine.

[41] Pike KN (1945) The intonation of American English. Ann Arbor: University of Michigan Press.

[42] Arvaniti E (2009) Rhythm, timing and the timing of rhythm. Phonetica 66: 46-63. doi:10.1159/000208930. PubMed: 19390230. 19390230 10.1159/000208930 PMC2790788

[43] Dauer RM (1983) Stress-timing and syllable-timing reanalyzed. J Phon 11: 51-62.

[44] Roach P (1982) On the distinction between “stress-timed” and “syllable-timed” languages. In: Crystal D Linguistic controversies: Essays in linguistic theory and practice in honour of FR Palmer. London: Edward Arnold pp. 73-79.

[45] Repp BH (1998) A microcosm of musical expression. I. Quantitative analysis of pianists’ timing in the initial measures of Chopin’s Etude in E major. J Acoust Soc Am 104: 1085-1100. doi:10.1121/1.423325. PubMed: 9714927. 9714927 10.1121/1.423325

[46] Cooper GW, Meyer LB (1960) The rhythmic structure of music. Chicago: University of Chicago Press.

[47] Selkirk EO (1984) Phonology and Syntax: The relation between sound and structure. Cambridge, MA: MIT Press.

[48] Lerdahl F (2001) The sounds of poetry viewed as music. Ann N Y Acad Sci 930: 337-354. PubMed: 11458840. 11458840 10.1111/j.1749-6632.2001.tb05743.x

[49] Lerdahl F (2003) The sounds of poetry viewed as music. In: Peretz I Zatorre RJ The cognitive neuroscience of music. Oxford: Oxford University Press pp. 413-429.

[50] Tillmann B, Dowling WJ (2007) Memory decreases for prose, but not for poetry. Mem Cogn 35(4): 628-639. doi:10.3758/BF03193301. 10.3758/bf03193301 17848021

[51] Cummins F (2009) Rhythm as an affordance for the entrainment of movement. Phonetica 66: 15-28. doi:10.1159/000208928. PubMed: 19390228. 19390228 10.1159/000208928

[52] Peelle JE, Davis MH (2012) Neural oscillations carry speech rhythm through to comprehension. Front Psychol 3: 320 PubMed: 22973251. 22973251 10.3389/fpsyg.2012.00320 PMC3434440

[53] Boltz MG, Jones MR (1986) Does rule recursion make melodies easier to reproduce? If not, what does? Cogn Psychol 18: 389-431. doi:10.1016/0010-0285(86)90005-8.

[54] Ellis RJ, Jones MR (2009) The role of accent salience and joint accent structure in meter perception. J Exp Psychol Hum Percept Perform 35(1): 264–280. doi:10.1037/a0013482. PubMed: 19170487. 19170487 10.1037/a0013482

[55] Jones MR (1987) Dynamic pattern structure in music: Recent theory and research. Percept Psychophys 41: 621–634. doi:10.3758/BF03210494. PubMed: 3615156. 3615156 10.3758/bf03210494

[56] Jones MR (1993) Dynamics of musical patterns: How do melody and rhythm fit together? In: Tighe TJ Dowling WJ Psychology and music: The understanding of melody and rhythm. Hillsdale, NJ: Erlbaum pp. 67-92.

[57] Jones MR, Pfordresher PQ (1997) Tracking melodic events using joint accent structure. Can J Exp Psychol 51: 271-291. doi:10.1037/1196-1961.51.4.271.

[58] Aschersleben G, Stenneken P, Cole J, Prinz W (2002) Timing mechanisms in sensorimotor synchronization. In: Prinz W Hommel B, Common mechanisms in perception and action, Attention and Performance XIX. New York: Oxford University Press pp. 227-244.

[59] Repp BH (2006) Musical synchronization. In: Altenmüller E Kesselring J Wiesendanger M Music, motor control, and the brain. Oxford: Oxford University Press pp. 55-76.

[60] Drake C, Jones MR, Baruch C (2000) The development of rhythmic attending in auditory sequences: Attunement, reference period, focal attending. Cognition 77: 251-288. doi:10.1016/S0010-0277(00)00106-2. PubMed: 11018511. 11018511 10.1016/s0010-0277(00)00106-2

[61] Villing RC, Repp BH, Ward TE, Timoney JM (2011) Measuring perceptual centers using the phase correction response. Atten. Percept Psychophys 73(5): 1614-1629. doi:10.3758/s13414-011-0110-1. 10.3758/s13414-011-0110-1 21431995

[62] Repp BH, Penel A (2002) Auditory dominance in temporal processing: new evidence from synchronization with simultaneous visual and auditory sequences. J Exp Psychol Hum Percept Perform 28: 1085-1099. doi:10.1037/0096-1523.28.5.1085. PubMed: 12421057. 12421057

[63] Repp BH, Penel A (2004) Rhythmic movement is attracted more strongly to auditory than to visual rhythms. Psychol Res 68: 252-270. PubMed: 12955504. 12955504 10.1007/s00426-003-0143-8

[64] Hove MJ, Iversen JR, Zhang A, Repp BH (2013) Synchronization with competing visual and auditory rhythms: Bouncing ball meets metronome. Psychol Res 77(4): 388-398. doi:10.1007/s00426-012-0441-0. PubMed: 22638726. 22638726 10.1007/s00426-012-0441-0

[65] Repp BH (2003) Phase attraction in sensorimotor synchronization with auditory sequences: Effects of single and periodic distractors on synchronization accuracy. J Exp Psychol Hum Percept Perform 29: 290-309. doi:10.1037/0096-1523.29.2.290. PubMed: 12760616. 12760616 10.1037/0096-1523.29.2.290

[66] Repp BH (2004) On the nature of phase attraction in sensorimotor synchronization with interleaved auditory sequences. Hum Mov Sci 23: 389-413. doi:10.1016/j.humov.2004.08.014. PubMed: 15541525. 15541525 10.1016/j.humov.2004.08.014

[67] Tuwim J (1980) Wiersze dla dzieci. Warszawa: Nasza Księgarnia.

[68] Brzechwa J (1980) Brzechwa dzieciom. Warszawa: Nasza Księgarnia.

[69] Boersma P, Weenink D (2008) Praat: Doing phonetics by computer. [Computer program, 15 July 2013 access]. Institute of Phonetic Sciences, University of Amsterdam. http://www.praat.org/.

[70] Boersma P (1993) Accurate short-term analysis of the fundamental frequency and the harmonics-to-noise ratio of a sampled sound. Proceedings of the Institute of Phonetic Sciences 17 University of Amsterdam . pp. 97-110

[71] Aschersleben G (2002) Temporal control of movements in sensorimotor synchronization. Brain Cogn 48(1): 66-79. doi:10.1006/brcg.2001.1304. PubMed: 11812033. 11812033 10.1006/brcg.2001.1304

[72] Biskupska M, Bruce D (1998) Łatwe piosenki na fortepian. Podkowa Leśna: Crescendo.

[73] Repp BH (2006) Does an auditory distractor sequence affect self-paced tapping? Acta Psychol 121: 81-107. doi:10.1016/j.actpsy.2005.06.006. PubMed: 16098944. 10.1016/j.actpsy.2005.06.006 16098944

[74] Corriveau KH, Pasquni ES, Goswami U (2007) Basic auditory processing skills and specific language impairment: A new look at an old hypothesis. J Speech Lang Hear Res 50: 1–20. 10.1044/1092-4388(2007/046) 17538107

[75] Huss M, Verney JP, Fosker T, Mead N, Goswami U (2011) Music, rhythm, rise time perception and developmental dyslexia: Perception of musical meter predicts reading and phonology. Cortex 47(6): 674-689. doi:10.1016/j.cortex.2010.07.010. PubMed: 20843509. 20843509 10.1016/j.cortex.2010.07.010

[76] Patel AD, Iversen JR, Rosenberg JC (2006) Comparing the rhythm and melody of speech and music: The case of British English and French. J Acoust Soc Am 119: 3034–3047. doi:10.1121/1.2179657. PubMed: 16708959. 16708959 10.1121/1.2179657

[77] Marie C, Magne C, Besson M (2011) Musicians and the metric structure of words. J Cogn Neurosci 23: 294–305. doi:10.1162/jocn.2010.21413. PubMed: 20044890. 20044890 10.1162/jocn.2010.21413

[78] Vorberg D, Wing A (1996) Modeling variability and dependence in timing. In: Heuer H Keele SW Handbook of perception and action (Vol. 2). London: Academic Press pp. 181-262.

[79] Pressing J (1999) The referential dynamics of cognition and action. Psychol Rev 106: 714-747. doi:10.1037/0033-295X.106.4.714.

[80] Vorberg D, Schulze H-H (2002) A two-level timing model for synchronization. J Math Psychol 46: 56-87. doi:10.1006/jmps.2001.1375.

[81] Aschersleben G, Bertelson P (2003) Temporal ventriloquism: Crossmodal interaction on the time dimension. 2. Evidence from sensorimotor synchronization. Int J Psychophysiol 50: 157–163. doi:10.1016/S0167-8760(03)00131-4. PubMed: 14511843. 14511843 10.1016/s0167-8760(03)00131-4

[82] Morein-Zamir S, Soto-Faraco S, Kingstone A (2003) Auditory capture of vision: Examining temporal ventriloquism. Cogn Brain Res 17: 154–163. doi:10.1016/S0926-6410(03)00089-2. PubMed: 12763201. 10.1016/s0926-6410(03)00089-2 12763201

[83] Kelso JAS, Zanone PG (2002) Coordination dynamics of learning and transfer across different effector systems. J Exp Psychol Hum Percept Perform 28: 776-797. doi:10.1037/0096-1523.28.4.776. PubMed: 12190250. 12190250

[84] Zanone PG, Kelso JAS (1997) Coordination dynamics of learning and transfer: Collective and component levels. J Exp Psychol Hum Percept Perform 23: 1454-1480. doi:10.1037/0096-1523.23.5.1454. PubMed: 9336961. 9336961 10.1037//0096-1523.23.5.1454

[85] Jones MR (2009) Musical time. In: Hallam S Cross I Thaut M The Oxford Handbook of Music Psychology. Oxford: Oxford University Press pp. 81-92.

[86] Jones MR (2010) Attending to sound patterns and the role of entrainment. In: Nobre C Coull JT Attention and time. New York: Oxford University Press pp. 317-330.

[87] Patel AD, Iversen JR, Chen Y, Repp BH (2005) The influence of metricality and modality on synchronization with a beat. Exp Brain Res 163: 226-238. doi:10.1007/s00221-004-2159-8. PubMed: 15654589. 15654589 10.1007/s00221-004-2159-8

[88] Chen JL, Zatorre RJ, Penhune VB (2006) Interactions between auditory and dorsal premotor cortex during synchronization to musical rhythms. NeuroImage 32: 1771-1781. doi:10.1016/j.neuroimage.2006.04.207. PubMed: 16777432. 16777432 10.1016/j.neuroimage.2006.04.207

[89] Chen JL, Penhune VB, Zatorre RJ (2008) Moving on time: Brain network for auditory-motor synchronization is modulated by rhythm complexity and musical training. J Cogn Neurosci 20(2): 226-239. doi:10.1162/jocn.2008.20018. PubMed: 18275331. 18275331 10.1162/jocn.2008.20018

[90] Dalla Bella S, Peretz I (2003) Congenital amusia interferes with the ability to synchronize with music. Ann N Y Acad Sci 999: 166-169. doi:10.1196/annals.1284.021. PubMed: 14681133. 14681133 10.1196/annals.1284.021

[91] Phillips-Silver J, Toiviainen P, Gosselin N, PIChE O, Nozaradan S et al (2011) Born to dance but beat-deaf: A new form of congenital amusia. Neuropsychologia 49: 961–969. doi:10.1016/j.neuropsychologia.2011.02.002. PubMed: 21316375. 21316375 10.1016/j.neuropsychologia.2011.02.002

[92] Sowiński J, Dalla Bella S (2013) Poor synchronization to the beat may result from deficient auditory-motor mapping, Neuropsychologia. http://dx.doi.org/10.1016/j.neuropsychologia.2013.06.027. 10.1016/j.neuropsychologia.2013.06.027 23838002

[93] Ramus F, Nespor M, Mehler J (1999) Correlates of linguistic rhythm in the speech signal. Cognition 73(3): 265-292. doi:10.1016/S0010-0277(99)00058-X. PubMed: 10585517. 10585517 10.1016/s0010-0277(99)00058-x

[94] Hayes B, Puppel S (1985) On the rhythm rule in Polish. In: van der Hulst H Smith N Advances in nonlinear phonology. Dordrecht: Foris Publications pp. 59-81.

